# Psychology and medical education: A historical perspective from the United States

**DOI:** 10.4103/0019-5545.37318

**Published:** 2007

**Authors:** Wade Pickren

**Affiliations:** Department of Psychology, Ryerson University, 350 Victoria Street, Room JOR 825 Toronto, Ontario, Canada

Psychology became an integral and popular part of the undergraduate curriculum in the United States by the 1920s. It continued to grow in popularity throughout the century and now, in the 21^st^ century, remains one of the top undergraduate degree programs in terms of the number of students who take it as their major. Psychology is also a popular and important program of undergraduate study for students who then pursue advanced education and training in a wide range of fields, including Medicine. Undergraduate Psychology courses that are popular with premedical students include Biological (Physiological) Psychology and Abnormal Psychology.

Even with the growth and popularity of Psychology and its increasing relevance for health care, there has been resistance to incorporating it in the training of physicians. For most of the 20^th^ century, Psychology was not considered a necessary part of medical education and psychologists were considered adjunctive in the practice of Medicine. However, by the last third of the 20^th^ century, the situation had begun to change. In this article, I describe efforts from early in the 20^th^ century to make Psychology a more formal part of medical education and suggest some reasons for why they failed to gain a purchase in the medical curriculum. I then briefly describe some factors that led to a greater acceptance of Psychology in medical training and settings and suggest some reasons for this acceptance. At the beginning of the 21^st^ century, lessons can be drawn from these examples to help articulate a viable role for psychologists in an integrated health-care system, both in India and in the United States.

## PSYCHOLOGY IN THE CONTEXT OF THE REFORM OF MEDICAL EDUCATION

In the first half of the 20^th^ century, there were many opportunities for interaction between psychologists and the medical profession in the United States.[1] In a few instances, there were genuine collaborations, such as in child guidance and neuroscience. In other arenas, such as mental testing and psychotherapy, physicians strongly resisted what they viewed as presumptuous incursions by psychologists into the domain of medicine.[2] Psychologists won the battle over mental testing in the United States, but physicians retained hegemony over psychotherapy until well after the end of World War II.[3] The question of whether instruction in Psychology should be part of the reformed medical curriculum was another area of debate.

The years around the turn of the 20^th^ century were a period of social change in American history. The social, political and professional order was transformed; new scientific disciplines were formed and professions were reformed. For scientific psychologists at the beginning of the 20^th^ century, one point of potential application for their discipline was through an alliance with the medical profession.

The practice of medicine was placed on a more scientific basis in the last quarter of the 19^th^ century. This resulted in greater success in the treatment of some diseases and a higher status for medicine in American society. The medical sciences became increasingly centered on the laboratory as the locus of their work.[4,5] Medical education began to focus on the several sciences considered basic to Medicine: anatomy, bacteriology, biochemistry, histology, pharmacology, physiology and pathology. This was the result of a gradual and halting reform of the medical school curriculum that began around 1840.[6] The reorganization of Harvard Medical School (1871) and the establishment of the Johns Hopkins University Medical School (1893) along lines emphasizing both clinical instruction and medical research were landmarks of the reform and signposts to the future of medical education. All the leading medical schools eventually raised standards of admission, and the length of training was expanded from two years to three years and then to four years.[7] The ideal of medical education became two years of preclinical instruction in the basic medical sciences, with laboratory work in the second year, followed by two years of clinical instruction.[8] In this context of curricular reform, questions about the putative role of "psychic" factors in disease, especially mental disease, led some physicians and psychologists to argue that medical students needed to be knowledgeable about psychology.

## SHOULD PSYCHOLOGY HAVE A PLACE IN THE MEDICAL CURRICULUM?

What did physicians think about psychology as part of medical training? Physicians who wrote about this issue wanted a psychology that was suited to their needs, practical and applicable to patient care. Perhaps a typical example of this came from George Dearborn of Tufts Medical School in the Boston, Massachusetts, area. What the medical curriculum needed from psychology, in Dearborn's view, was two things: 1) an explication of normal mental processes so that a physician could recognize a deviation from the normal and 2) an explanation of the psychological factors in the relationship of doctor and patient, especially as this affects disease and recovery from disease. Dearborn believed that a psychology able to make these contributions would be welcomed into the reformed medical curriculum.[9,10]

What did psychologists think about psychology in the training of physicians? Psychologists who addressed this issue typically wanted to introduce some version of standard experimental psychology into the training of medical students, while also contending that psychology could benefit both physicians and their patients by helping them understand the psychological aspects of daily life.[11]

### The key years of the debate: 1911-1918

After the Flexner report was published in 1910, there were several years of intense debate and struggle over the medical curriculum. It was in this period that psychologists made their strongest case for inclusion of psychology in the curriculum. Despite their efforts, psychology did not become a basic course of instruction at this time.

The medical and psychological literature of the time had numerous articles pro and con in the debate over inclusion. For example, a special session on the issue was held at the 1911 meeting of the American Psychological Association (APA), with psychologists, psychiatrists and neurologists involved.[12] All agreed that psychology merited inclusion in the training of physicians, although each one expressed different views about the place of psychology in the medical curriculum. The physicians wanted medical control over the content of psychological instruction to insure that it was a practical psychology oriented to the needs of physicians and, especially, the needs of those who wanted to specialize in Psychiatry. The psychologists argued for a normal psychology, taught by psychologists, oriented around what were then the discipline's traditional subjects: habit-formation, association, emotion, fatigue and memory. Sessions like this were a fine opportunity that could have raised the visibility and stature of American psychology, especially because leading psychiatrists were also interested in placing more emphasis on psychological topics in medical training.

The issue was important enough that surveys were conducted to ascertain what was being done in medical education vis-à-vis psychology. In 1913, E. Stanley Abbot surveyed 85 medical schools. Only four schools required a course in normal psychology, while thirty ignored it altogether. Two schools required a course in abnormal psychology and two offered such a course as an elective. Eleven schools offered instruction in psychology in other courses, while seven planned to do so. Abbot found that the total number of schools requiring, advising or teaching psychology was 26 out of the 58 that replied. Of course, as Abbot noted, these numbers did not indicate the type, the quality or the quantity of psychological instruction. They did indicate that psychology was not ignored in the competition for curricular space. What was clear from this survey was that psychology was not considered basic to medical training.[13]

The APA formed a Committee on Psychology and Medical Education that sent out a survey in 1913 to medical schools in order to discover interest in psychology, the amount of instruction currently given to psychological topics, what amount, if any, of such instruction was planned for the near future and who did the instruction (psychologists or physicians). The Committee received replies from 71 out of 114 medical schools. Overall, the results were discouraging for psychologists. Few schools offered instruction in psychology, although ten planned to do so in the near future. Additionally, there appeared to be great confusion on the part of those replying as to what was meant by psychology, which reflected the confusion within the discipline itself. The one encouraging note came from the data from the highest-ranked schools. Several of these schools already offered instruction in some form in psychology, while several more planned to do so.[11]

Psychologist Fred Wells argued that the onus was on psychology to provide medical students with “something they can use.” Medicine, he pointed out, deals with problems of human suffering, while psychology is concerned with scientific investigation of psychological phenomena not necessarily related to medical problems. Unlike most other psychologists who wrote about this issue, Wells argued that it was useless for psychologists to expect medical schools to include a psychology that was not oriented to the practical needs of medicine.[14]

## WHY DID PSYCHOLOGY FAIL TO BE INCLUDED IN THE MEDICAL CURRICULUM?

When seen within the larger context of the reform of medical education and the contested content of the medical curriculum, the suggestions of psychologists and psychiatrists can be understood as united in their plea for a place for psychology in the medical curriculum. These participants all were invested in the importance of psychological instruction in the medical school.

Why then did they not succeed? Two reasons stand out. First, psychologists and their partners in psychiatry and neurology were marginal to the leaders of medical education. At the time, psychiatrists and neurologists were not as well respected as they later became. Psychologists and their allies were bargaining from a position of weakness in the debate over the extension of the medical school curriculum to include psychology. The medical reform leaders, like Abraham Flexner and William Henry Welch, did not see psychology as crucial to medical science or practice.[15] When the medical curriculum finally settled, the competition proved too fierce for psychology. Secondarily, although psychologists and their physician partners agreed that medical students would benefit from a practical psychology, the psychologists were unable to articulate a version of psychological science that was appealing enough either to their allies or to leading medical reformers. Psychologists tended to emphasize the necessity of exposure to experimental psychology and its laboratory methods and the importance of instruction by psychologists. The differences between the psychologists and the physicians were great enough to undermine psychologists' arguments for the inclusion of psychology in the medical curriculum.

The role of psychology in medical training in the USA did become a reality much later in the 20^th^ century.[16,17] What facilitated the formal inclusion of psychology in medical education was the emergence of proven psychological expertise in health care and a demonstrated utility for psychological approaches to issues that were of significant concern to the medical profession.

As psychologists developed sound clinical skills in the years after World War II, they were able to gradually add clinical contributions to their research work in health and disease.[18] In settings as diverse as medical schools, general hospitals, psychiatry departments, Veterans Affairs hospitals and many others, psychologists contributed to the understanding of health and disease.[19] The specialty of Health Psychology became an APA Division in 1978 and was a strong indicator of the role of psychology and psychologists in the provision of an high-quality health-care delivery.[20]

After the early failure to find a place in formal medical training, psychologists took advantage of the opportunities that emerged from their past experiences to move in the new directions that emerged as the social and intellectual ecosystem of which they were a part shifted and changed. In the current flux over the provision of health care, new opportunities exist for psychologists as a vital part of an integrated health-care system. If psychologists are able to learn from their own history how to forge effective alliances and how to engage other health-care professions and health policy makers from a position of demonstrated excellence, then the future is bright for psychology in health care, whether in the United States or in India.
